# 4-(4-Bromo­phen­yl)-6-(1*H*-indol-3-yl)-2,2′-bipyridine-5-carbonitrile

**DOI:** 10.1107/S1600536809001354

**Published:** 2009-02-04

**Authors:** P. Ramesh, A. Subbiahpandi, P. Thirumurugan, Paramasivan T. Perumal, M. N. Ponnuswamy

**Affiliations:** a Department of Physics, Presidency College (Autonomous), Chennai 600 005, India; b Organic Chemistry Division, Central Leather Research Institute, Adyar, Chennai 600 020, India; c Centre of Advanced Study in Crystallography and Biophysics, University of Madras, Guindy Campus, Chennai 600 025, India

## Abstract

In the title compound, C_25_H_15_BrN_4_, the two pyridine rings lie in a common plane [r.m.s. deviation = 0.023 (2) Å], whereas the bromo­phenyl and indole rings are twisted away from this plane by 52.82 (12) and 28.02 (10)°, respectively. The crystal structure is stabilized by inter­molecular N—H⋯N inter­actions.

## Related literature

Compounds having an indole ring system have been shown to display high aldose reductase inhibitory activity (Rajeswaran *et al.*, 1999 ▶). For hydrogen-bond motifs, see: Bernstein *et al.* (1995 ▶);
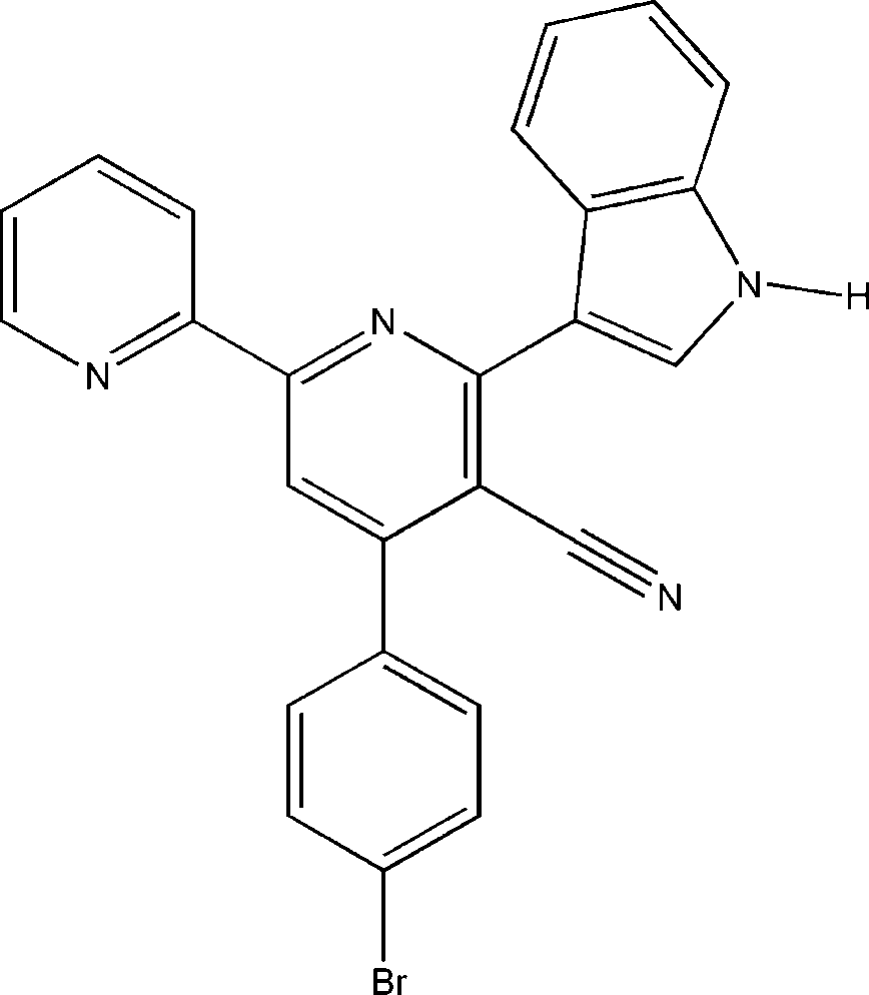


## Experimental

### 

#### Crystal data

C_25_H_15_BrN_4_
*M* *_r_* = 451.32
Orthorhombic, 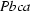

*a* = 14.7393 (4) Å
*b* = 10.7465 (3) Å
*c* = 25.4251 (7) Å
*V* = 4027.23 (19) Å^3^
*Z* = 8
Mo *K*α radiation
μ = 2.06 mm^−1^
*T* = 293 (2) K
0.29 × 0.26 × 0.22 mm

#### Data collection

Bruker Kappa APEXII area-detector diffractometer
Absorption correction: multi-scan (*SADABS*; Sheldrick, 2001 ▶) *T*_min_ = 0.556, *T*_max_ = 0.635
47903 measured reflections
5545 independent reflections
3138 reflections with *I* > 2σ(*I*)
*R*_int_ = 0.057

#### Refinement

*R*[*F* ^2^ > 2σ(*F* ^2^)] = 0.040
*wR*(*F* ^2^) = 0.120
*S* = 0.99
5545 reflections
272 parameters
H-atom parameters constrained
Δρ_max_ = 0.42 e Å^−3^
Δρ_min_ = −0.58 e Å^−3^

### 

Data collection: *APEX2* (Bruker, 2004 ▶); cell refinement: *SAINT* (Bruker, 2004 ▶); data reduction: *SAINT*; program(s) used to solve structure: *SHELXS97* (Sheldrick, 2008 ▶); program(s) used to refine structure: *SHELXL97* (Sheldrick, 2008 ▶); molecular graphics: *ORTEP-3* (Farrugia, 1997 ▶); software used to prepare material for publication: *SHELXL97* and *PLATON* (Spek, 2003 ▶).

## Supplementary Material

Crystal structure: contains datablocks global, I. DOI: 10.1107/S1600536809001354/bt2842sup1.cif

Structure factors: contains datablocks I. DOI: 10.1107/S1600536809001354/bt2842Isup2.hkl

Additional supplementary materials:  crystallographic information; 3D view; checkCIF report

## Figures and Tables

**Table 1:** Hydrogen-bond geometry (Å, °)

*D*—H⋯*A*	*D*—H	H⋯*A*	*D*⋯*A*	*D*—H⋯*A*
N14—H14⋯N17^i^	0.86	2.22	2.980 (3)	147

Symmetry code: (i) 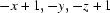.

**Fig. 1.**
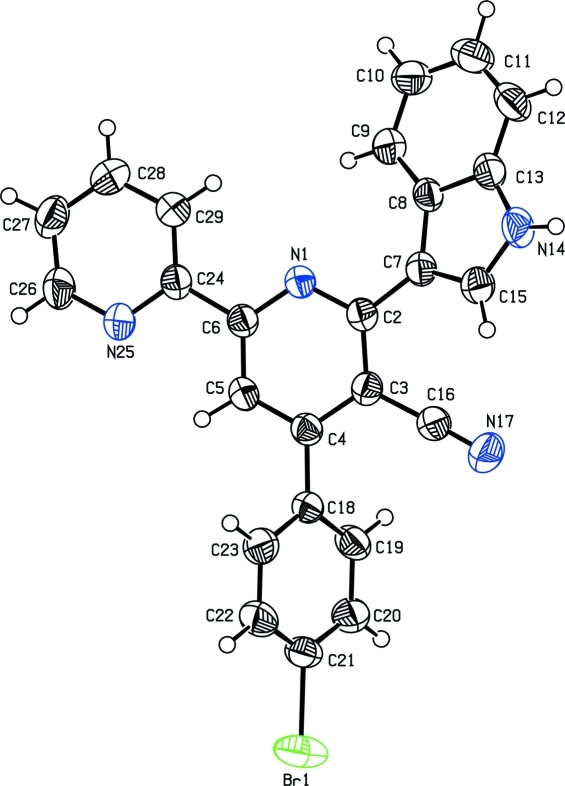
Perspective view of the molecule showing the displacement ellipsoids at 50% probability level. The H atoms are shown as small circles of arbitrary radii.

**Fig. 2.**
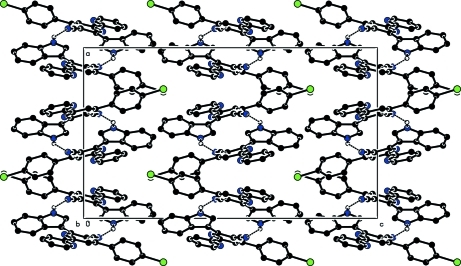
The crystal packing of the molecules viewed down the b axis. H atoms not involved in hydrogen bonding are omitted for clarity.

## supplementary crystallographic information

### Comment

Compounds having indole ring system are proved to display high aldose reductase inhibitory activity (Rajeswaran *et al.*, 1999). Against this background and to ascertain the detailed conformation, the crystal structure determination of the title compound has been carried out.

The *ORTEP* diagram of the title compound is shown in Fig. 1. The two pyridine rings lie in the same plane as can be seen from the dihedral angle of 3.61 (13)°. The bromophenyl and indole rings are twisted away from the bipyridine ring by 52.82 (12)° and 28.02 (10)°, respectively. The sum of the bond angles at N14 (360.0°) in the indole ring is in accordance with *sp^2^* hybridization. The bond angle of C3—C16—N17 [178.4 (3)°] shows the linearity of the cyano group, a feature observed in carbonitrile compounds.

The crystal packing is controlled by C—H···N intermolecular interactions in addition to van der Waals forces. Atom N14 (*x*, *y*, *z*) donates one proton to N17 at (-*x* + 1,-*y*,-*z* + 1) which connects the molecules to form a *R*_2_^2^ (16) dimer (Bernstein *et al.*, 1995).

### Experimental

A mixture of 3-cyanoacetyl indole (1 mmol), 4-bromobenzaldehyde (1 mmol) and 2-acetyl pyridine (1 mmol) in 5 gm of ammonium acetate under neat condition was refluxed 6–8 hrs. After the completion of the reaction (as monitored by TLC), it was poured into water and extracted with ethyl acetate. The organic layer was dried over sodium sulfate and concentrated under vacuo. The crude product was chromatographed and isolated in 80% yield (90:10, petroleum ether: ethyl acetate) and recrystallized in ethanol.

### Refinement

H atoms were positioned geometrically (N—H=0.86 Å, and C—H=0.93 Å) and allowed to ride on their parent atoms, with 1.2*U*_eq_(C,N).

### Crystal data

**Table:**

C_25_H_15_BrN_4_	*F*(000) = 1824
*M**_r_* = 451.32	*D*_x_ = 1.489 Mg m^−^^3^
Orthorhombic, *P**b**c**a*	Mo *K*α radiation, λ = 0.71073 Å
Hall symbol: -P 2ac 2ab	Cell parameters from 5842 reflections
*a* = 14.7393 (4) Å	θ = 1.6–29.4°
*b* = 10.7465 (3) Å	µ = 2.06 mm^−^^1^
*c* = 25.4251 (7) Å	*T* = 293 K
*V* = 4027.23 (19) Å^3^	Block, colorless
*Z* = 8	0.29 × 0.26 × 0.22 mm

### Data collection

**Table:**

Bruker Kappa APEXII area-detector diffractometer	5545 independent reflections
Radiation source: fine-focus sealed tube	3138 reflections with *I* > 2σ(*I*)
graphite	*R*_int_ = 0.057
ω and φ scans	θ_max_ = 29.4°, θ_min_ = 1.6°
Absorption correction: multi-scan (*SADABS*; Sheldrick, 2001)	*h* = −17→20
*T*_min_ = 0.556, *T*_max_ = 0.635	*k* = −14→14
47903 measured reflections	*l* = −35→35

### Refinement

**Table:**

Refinement on *F*^2^	Secondary atom site location: difference Fourier map
Least-squares matrix: full	Hydrogen site location: inferred from neighbouring sites
*R*[*F*^2^ > 2σ(*F*^2^)] = 0.040	H-atom parameters constrained
*wR*(*F*^2^) = 0.120	*w* = 1/[σ^2^(*F*_o_^2^) + (0.0524*P*)^2^ + 1.6981*P*] where *P* = (*F*_o_^2^ + 2*F*_c_^2^)/3
*S* = 0.99	(Δ/σ)_max_ = 0.001
5545 reflections	Δρ_max_ = 0.42 e Å^−^^3^
272 parameters	Δρ_min_ = −0.58 e Å^−^^3^
0 restraints	Extinction correction: *SHELXL97* (Sheldrick, 2008), Fc^*^=kFc[1+0.001xFc^2^λ^3^/sin(2θ)]^-1/4^
Primary atom site location: structure-invariant direct methods	Extinction coefficient: 0.0015 (2)

### Special details

**Table:**

Geometry. All e.s.d.'s (except the e.s.d. in the dihedral angle between two l.s. planes) are estimated using the full covariance matrix. The cell e.s.d.'s are taken into account individually in the estimation of e.s.d.'s in distances, angles and torsion angles; correlations between e.s.d.'s in cell parameters are only used when they are defined by crystal symmetry. An approximate (isotropic) treatment of cell e.s.d.'s is used for estimating e.s.d.'s involving l.s. planes.
Refinement. Refinement of *F*^2^ against ALL reflections. The weighted *R*-factor *wR* and goodness of fit *S* are based on *F*^2^, conventional *R*-factors *R* are based on *F*, with *F* set to zero for negative *F*^2^. The threshold expression of *F*^2^ > σ(*F*^2^) is used only for calculating *R*-factors(gt) *etc*. and is not relevant to the choice of reflections for refinement. *R*-factors based on *F*^2^ are statistically about twice as large as those based on *F*, and *R*- factors based on ALL data will be even larger.

### Fractional atomic coordinates and isotropic or equivalent isotropic displacement parameters (Å^2^)

**Table:**

	*x*	*y*	*z*	*U*_iso_*/*U*_eq_	
Br1	0.24258 (2)	0.41762 (4)	0.228490 (11)	0.07539 (16)	
N1	0.40577 (13)	0.47359 (16)	0.56512 (7)	0.0397 (4)	
C2	0.41263 (15)	0.3612 (2)	0.54258 (9)	0.0385 (5)	
C3	0.38681 (16)	0.3440 (2)	0.48975 (9)	0.0399 (5)	
C4	0.35912 (16)	0.4449 (2)	0.45926 (9)	0.0391 (5)	
C5	0.35655 (16)	0.5595 (2)	0.48331 (9)	0.0426 (5)	
H5	0.3406	0.6298	0.4641	0.051*	
C6	0.37776 (15)	0.5701 (2)	0.53619 (9)	0.0387 (5)	
C7	0.44948 (15)	0.2632 (2)	0.57570 (9)	0.0400 (5)	
C8	0.44917 (15)	0.2595 (2)	0.63245 (9)	0.0402 (5)	
C9	0.41165 (18)	0.3326 (2)	0.67203 (10)	0.0490 (6)	
H9	0.3769	0.4021	0.6637	0.059*	
C10	0.4265 (2)	0.3010 (3)	0.72342 (10)	0.0591 (7)	
H10	0.4022	0.3503	0.7499	0.071*	
C11	0.4771 (2)	0.1972 (3)	0.73681 (11)	0.0640 (8)	
H11	0.4861	0.1783	0.7721	0.077*	
C12	0.5139 (2)	0.1223 (3)	0.69920 (11)	0.0596 (7)	
H12	0.5473	0.0521	0.7082	0.072*	
C13	0.49973 (16)	0.1545 (2)	0.64702 (10)	0.0463 (6)	
N14	0.52963 (15)	0.09858 (18)	0.60195 (9)	0.0533 (5)	
H14	0.5626	0.0327	0.6007	0.064*	
C15	0.49954 (16)	0.1625 (2)	0.55983 (10)	0.0485 (6)	
H15	0.5111	0.1413	0.5250	0.058*	
C16	0.38817 (18)	0.2223 (2)	0.46641 (10)	0.0480 (6)	
N17	0.38837 (19)	0.1271 (2)	0.44684 (10)	0.0694 (7)	
C18	0.33250 (16)	0.4340 (2)	0.40326 (9)	0.0403 (5)	
C19	0.26769 (17)	0.3498 (2)	0.38682 (10)	0.0504 (6)	
H19	0.2413	0.2960	0.4110	0.060*	
C20	0.24170 (18)	0.3450 (2)	0.33465 (11)	0.0530 (7)	
H20	0.1982	0.2879	0.3237	0.064*	
C21	0.28003 (18)	0.4241 (2)	0.29947 (10)	0.0481 (6)	
C22	0.34485 (19)	0.5078 (3)	0.31444 (10)	0.0562 (7)	
H22	0.3712	0.5608	0.2899	0.067*	
C23	0.37058 (19)	0.5126 (2)	0.36651 (10)	0.0523 (6)	
H23	0.4143	0.5697	0.3770	0.063*	
C24	0.36885 (16)	0.6912 (2)	0.56355 (9)	0.0410 (5)	
N25	0.33924 (15)	0.78608 (18)	0.53415 (8)	0.0516 (5)	
C26	0.32987 (19)	0.8957 (2)	0.55752 (12)	0.0562 (7)	
H26	0.3101	0.9624	0.5373	0.067*	
C27	0.3474 (2)	0.9165 (2)	0.60946 (12)	0.0595 (7)	
H27	0.3388	0.9947	0.6243	0.071*	
C28	0.3779 (3)	0.8194 (3)	0.63905 (12)	0.0756 (9)	
H28	0.3908	0.8304	0.6746	0.091*	
C29	0.3895 (2)	0.7051 (2)	0.61584 (10)	0.0624 (8)	
H29	0.4110	0.6381	0.6353	0.075*	

### Atomic displacement parameters (Å^2^)

**Table:**

	*U*^11^	*U*^22^	*U*^33^	*U*^12^	*U*^13^	*U*^23^
Br1	0.0741 (2)	0.1126 (3)	0.03942 (16)	0.02193 (18)	−0.00909 (13)	0.00200 (15)
N1	0.0451 (11)	0.0356 (10)	0.0385 (10)	0.0036 (8)	0.0000 (8)	0.0014 (8)
C2	0.0390 (13)	0.0360 (12)	0.0403 (12)	0.0018 (9)	0.0016 (10)	0.0024 (10)
C3	0.0419 (13)	0.0357 (12)	0.0420 (13)	0.0005 (10)	0.0031 (10)	−0.0014 (10)
C4	0.0422 (13)	0.0380 (12)	0.0370 (12)	0.0002 (10)	0.0033 (10)	0.0030 (10)
C5	0.0528 (14)	0.0342 (12)	0.0408 (13)	0.0033 (10)	−0.0002 (10)	0.0050 (10)
C6	0.0411 (12)	0.0357 (11)	0.0395 (12)	0.0005 (9)	0.0029 (10)	0.0020 (10)
C7	0.0410 (13)	0.0346 (11)	0.0444 (13)	0.0009 (10)	−0.0015 (10)	0.0008 (10)
C8	0.0428 (13)	0.0337 (11)	0.0442 (13)	−0.0045 (10)	−0.0066 (10)	0.0054 (10)
C9	0.0578 (16)	0.0416 (13)	0.0477 (14)	−0.0040 (12)	0.0001 (12)	0.0020 (11)
C10	0.0737 (19)	0.0608 (17)	0.0427 (15)	−0.0128 (14)	−0.0027 (13)	0.0005 (12)
C11	0.074 (2)	0.0704 (19)	0.0475 (15)	−0.0201 (16)	−0.0152 (14)	0.0149 (14)
C12	0.0648 (18)	0.0527 (15)	0.0613 (17)	−0.0053 (13)	−0.0194 (14)	0.0189 (14)
C13	0.0463 (14)	0.0390 (13)	0.0537 (15)	−0.0028 (10)	−0.0091 (12)	0.0059 (11)
N14	0.0550 (13)	0.0400 (11)	0.0648 (14)	0.0130 (10)	−0.0099 (11)	0.0043 (10)
C15	0.0511 (15)	0.0430 (13)	0.0513 (14)	0.0073 (11)	−0.0027 (12)	−0.0010 (11)
C16	0.0553 (15)	0.0438 (14)	0.0450 (14)	0.0084 (11)	−0.0056 (11)	−0.0008 (11)
N17	0.0931 (19)	0.0476 (13)	0.0675 (16)	0.0163 (12)	−0.0173 (14)	−0.0139 (12)
C18	0.0479 (14)	0.0375 (12)	0.0354 (11)	0.0036 (10)	0.0013 (10)	0.0011 (10)
C19	0.0563 (16)	0.0490 (14)	0.0458 (14)	−0.0095 (12)	−0.0059 (12)	0.0126 (11)
C20	0.0556 (16)	0.0533 (15)	0.0502 (15)	−0.0060 (12)	−0.0131 (12)	0.0009 (12)
C21	0.0544 (15)	0.0538 (14)	0.0360 (12)	0.0118 (13)	−0.0038 (11)	0.0011 (11)
C22	0.0705 (18)	0.0571 (16)	0.0412 (14)	−0.0020 (14)	0.0107 (13)	0.0090 (12)
C23	0.0626 (17)	0.0472 (14)	0.0472 (14)	−0.0133 (12)	0.0042 (12)	0.0015 (12)
C24	0.0433 (13)	0.0354 (12)	0.0442 (13)	0.0003 (10)	0.0044 (10)	0.0021 (10)
N25	0.0663 (14)	0.0373 (10)	0.0511 (13)	0.0095 (10)	−0.0024 (11)	−0.0019 (9)
C26	0.0650 (18)	0.0387 (13)	0.0648 (18)	0.0103 (12)	0.0007 (14)	0.0007 (12)
C27	0.0716 (18)	0.0421 (14)	0.0647 (18)	0.0018 (13)	0.0102 (14)	−0.0117 (13)
C28	0.122 (3)	0.0565 (18)	0.0484 (17)	0.0025 (18)	−0.0037 (17)	−0.0114 (14)
C29	0.099 (2)	0.0438 (14)	0.0446 (15)	0.0048 (14)	−0.0079 (15)	0.0001 (12)

### Geometric parameters (Å, °)

**Table:**

Br1—C21	1.888 (2)	N14—C15	1.347 (3)
N1—C6	1.336 (3)	N14—H14	0.8600
N1—C2	1.341 (3)	C15—H15	0.9300
C2—C3	1.408 (3)	C16—N17	1.138 (3)
C2—C7	1.453 (3)	C18—C23	1.379 (3)
C3—C4	1.394 (3)	C18—C19	1.381 (3)
C3—C16	1.437 (3)	C19—C20	1.382 (3)
C4—C5	1.376 (3)	C19—H19	0.9300
C4—C18	1.482 (3)	C20—C21	1.357 (4)
C5—C6	1.385 (3)	C20—H20	0.9300
C5—H5	0.9300	C21—C22	1.367 (4)
C6—C24	1.481 (3)	C22—C23	1.378 (3)
C7—C15	1.371 (3)	C22—H22	0.9300
C7—C8	1.443 (3)	C23—H23	0.9300
C8—C9	1.391 (3)	C24—N25	1.338 (3)
C8—C13	1.403 (3)	C24—C29	1.372 (3)
C9—C10	1.368 (3)	N25—C26	1.327 (3)
C9—H9	0.9300	C26—C27	1.364 (4)
C10—C11	1.383 (4)	C26—H26	0.9300
C10—H10	0.9300	C27—C28	1.363 (4)
C11—C12	1.363 (4)	C27—H27	0.9300
C11—H11	0.9300	C28—C29	1.373 (4)
C12—C13	1.387 (3)	C28—H28	0.9300
C12—H12	0.9300	C29—H29	0.9300
C13—N14	1.367 (3)		
			
C6—N1—C2	119.15 (19)	C13—N14—H14	125.2
N1—C2—C3	120.32 (19)	N14—C15—C7	110.2 (2)
N1—C2—C7	115.66 (19)	N14—C15—H15	124.9
C3—C2—C7	124.0 (2)	C7—C15—H15	124.9
C4—C3—C2	120.5 (2)	N17—C16—C3	178.4 (3)
C4—C3—C16	118.8 (2)	C23—C18—C19	118.5 (2)
C2—C3—C16	120.6 (2)	C23—C18—C4	119.7 (2)
C5—C4—C3	117.2 (2)	C19—C18—C4	121.7 (2)
C5—C4—C18	119.4 (2)	C18—C19—C20	120.5 (2)
C3—C4—C18	123.4 (2)	C18—C19—H19	119.8
C4—C5—C6	119.9 (2)	C20—C19—H19	119.8
C4—C5—H5	120.0	C21—C20—C19	119.6 (2)
C6—C5—H5	120.0	C21—C20—H20	120.2
N1—C6—C5	122.7 (2)	C19—C20—H20	120.2
N1—C6—C24	116.8 (2)	C20—C21—C22	121.3 (2)
C5—C6—C24	120.5 (2)	C20—C21—Br1	119.0 (2)
C15—C7—C8	105.9 (2)	C22—C21—Br1	119.64 (19)
C15—C7—C2	127.0 (2)	C21—C22—C23	119.0 (2)
C8—C7—C2	126.7 (2)	C21—C22—H22	120.5
C9—C8—C13	118.3 (2)	C23—C22—H22	120.5
C9—C8—C7	135.1 (2)	C22—C23—C18	121.1 (2)
C13—C8—C7	106.5 (2)	C22—C23—H23	119.5
C10—C9—C8	119.1 (2)	C18—C23—H23	119.5
C10—C9—H9	120.4	N25—C24—C29	122.0 (2)
C8—C9—H9	120.4	N25—C24—C6	115.9 (2)
C9—C10—C11	121.4 (3)	C29—C24—C6	122.1 (2)
C9—C10—H10	119.3	C26—N25—C24	117.4 (2)
C11—C10—H10	119.3	N25—C26—C27	124.0 (2)
C12—C11—C10	121.2 (3)	N25—C26—H26	118.0
C12—C11—H11	119.4	C27—C26—H26	118.0
C10—C11—H11	119.4	C28—C27—C26	118.1 (2)
C11—C12—C13	117.6 (3)	C28—C27—H27	120.9
C11—C12—H12	121.2	C26—C27—H27	120.9
C13—C12—H12	121.2	C27—C28—C29	119.2 (3)
N14—C13—C12	130.1 (2)	C27—C28—H28	120.4
N14—C13—C8	107.7 (2)	C29—C28—H28	120.4
C12—C13—C8	122.2 (2)	C24—C29—C28	119.1 (3)
C15—N14—C13	109.6 (2)	C24—C29—H29	120.4
C15—N14—H14	125.2	C28—C29—H29	120.4
			
C6—N1—C2—C3	−2.8 (3)	C7—C8—C13—C12	179.7 (2)
C6—N1—C2—C7	175.9 (2)	C12—C13—N14—C15	−179.7 (3)
N1—C2—C3—C4	4.0 (3)	C8—C13—N14—C15	−0.7 (3)
C7—C2—C3—C4	−174.6 (2)	C13—N14—C15—C7	0.5 (3)
N1—C2—C3—C16	−175.8 (2)	C8—C7—C15—N14	−0.2 (3)
C7—C2—C3—C16	5.6 (4)	C2—C7—C15—N14	173.8 (2)
C2—C3—C4—C5	−1.3 (3)	C4—C3—C16—N17	−4(11)
C16—C3—C4—C5	178.4 (2)	C2—C3—C16—N17	176 (100)
C2—C3—C4—C18	179.0 (2)	C5—C4—C18—C23	50.8 (3)
C16—C3—C4—C18	−1.3 (3)	C3—C4—C18—C23	−129.5 (3)
C3—C4—C5—C6	−2.3 (3)	C5—C4—C18—C19	−126.8 (3)
C18—C4—C5—C6	177.4 (2)	C3—C4—C18—C19	52.9 (3)
C2—N1—C6—C5	−1.0 (3)	C23—C18—C19—C20	−0.1 (4)
C2—N1—C6—C24	178.2 (2)	C4—C18—C19—C20	177.5 (2)
C4—C5—C6—N1	3.6 (4)	C18—C19—C20—C21	−0.3 (4)
C4—C5—C6—C24	−175.5 (2)	C19—C20—C21—C22	0.8 (4)
N1—C2—C7—C15	−149.1 (2)	C19—C20—C21—Br1	−179.2 (2)
C3—C2—C7—C15	29.6 (4)	C20—C21—C22—C23	−0.8 (4)
N1—C2—C7—C8	23.7 (3)	Br1—C21—C22—C23	179.1 (2)
C3—C2—C7—C8	−157.7 (2)	C21—C22—C23—C18	0.4 (4)
C15—C7—C8—C9	−179.9 (3)	C19—C18—C23—C22	0.0 (4)
C2—C7—C8—C9	6.1 (4)	C4—C18—C23—C22	−177.7 (2)
C15—C7—C8—C13	−0.3 (3)	N1—C6—C24—N25	−178.9 (2)
C2—C7—C8—C13	−174.2 (2)	C5—C6—C24—N25	0.3 (3)
C13—C8—C9—C10	1.1 (4)	N1—C6—C24—C29	1.0 (4)
C7—C8—C9—C10	−179.3 (3)	C5—C6—C24—C29	−179.8 (3)
C8—C9—C10—C11	−0.8 (4)	C29—C24—N25—C26	−0.3 (4)
C9—C10—C11—C12	−0.1 (4)	C6—C24—N25—C26	179.5 (2)
C10—C11—C12—C13	0.7 (4)	C24—N25—C26—C27	−0.9 (4)
C11—C12—C13—N14	178.6 (3)	N25—C26—C27—C28	1.2 (5)
C11—C12—C13—C8	−0.3 (4)	C26—C27—C28—C29	−0.2 (5)
C9—C8—C13—N14	−179.7 (2)	N25—C24—C29—C28	1.2 (4)
C7—C8—C13—N14	0.6 (3)	C6—C24—C29—C28	−178.7 (3)
C9—C8—C13—C12	−0.6 (4)	C27—C28—C29—C24	−0.9 (5)

### Hydrogen-bond geometry (Å, °)

**Table:**

*D*—H···*A*	*D*—H	H···*A*	*D*···*A*	*D*—H···*A*
N14—H14···N17^i^	0.86	2.22	2.980 (3)	147

Symmetry codes: (i) −*x*+1, −*y*, −*z*+1.
